# Online Social Networking and Addiction—A Review of the Psychological Literature

**DOI:** 10.3390/ijerph8093528

**Published:** 2011-08-29

**Authors:** Daria J. Kuss, Mark D. Griffiths

**Affiliations:** International Gaming Research Unit, Psychology Division, Nottingham Trent University, NG1 4BU, UK; E-Mail: mark.griffiths@ntu.ac.uk

**Keywords:** social network addiction, social networking sites, literature review, motivations, personality, negative consequences, comorbidity, specificity

## Abstract

Social Networking Sites (SNSs) are virtual communities where users can create individual public profiles, interact with real-life friends, and meet other people based on shared interests. They are seen as a ‘global consumer phenomenon’ with an exponential rise in usage within the last few years. Anecdotal case study evidence suggests that ‘addiction’ to social networks on the Internet may be a potential mental health problem for some users. However, the contemporary scientific literature addressing the addictive qualities of social networks on the Internet is scarce. Therefore, this literature review is intended to provide empirical and conceptual insight into the emerging phenomenon of addiction to SNSs by: (1) outlining SNS usage patterns, (2) examining motivations for SNS usage, (3) examining personalities of SNS users, (4) examining negative consequences of SNS usage, (5) exploring potential SNS addiction, and (6) exploring SNS addiction specificity and comorbidity. The findings indicate that SNSs are predominantly used for social purposes, mostly related to the maintenance of established offline networks. Moreover, extraverts appear to use social networking sites for social enhancement, whereas introverts use it for social compensation, each of which appears to be related to greater usage, as does low conscientiousness and high narcissism. Negative correlates of SNS usage include the decrease in real life social community participation and academic achievement, as well as relationship problems, each of which may be indicative of potential addiction.

## 1. Introduction

*“I’m an addict. I just get lost in Facebook”* replies a young mother when asked why she does not see herself able to help her daughter with her homework. Instead of supporting her child, she spends her time chatting and browsing the social networking site [1]. This case, while extreme, is suggestive of a potential new mental health problem that emerges as Internet social networks proliferate. Newspaper stories have also reported similar cases, suggesting that the popular press was early to discern the potentially addictive qualities of social networking sites (SNS; *i.e.*, [2,3]). Such media coverage has alleged that women are at greater risk than men for developing addictions to SNSs [4].

The mass appeal of social networks on the Internet could potentially be a cause for concern, particularly when attending to the gradually increasing amounts of time people spend online [5]. On the Internet, people engage in a variety of activities some of which may be potentially to be addictive. Rather than becoming addicted to the medium *per se,* some users may develop an addiction to specific activities they carry out online [6]. Specifically, Young [7] argues that there are five different types of internet addiction, namely *computer addiction* (*i.e.*, computer game addiction), *information overload* (*i.e.*, web surfing addiction), *net compulsions* (*i.e.*, online gambling or online shopping addiction), *cybersexual addiction* (*i.e.*, online pornography or online sex addiction), and *cyber-relationship addiction* (*i.e.*, an addiction to online relationships). SNS addiction appears to fall in the last category since the purpose and main motivation to use SNSs is to establish and maintain both on- and offline relationships (for a more detailed discussion of this please refer to the section on motivations for SNS usage). From a clinical psychologist’s perspective, it may be plausible to speak specifically of ‘*Facebook* Addiction Disorder’ (or more generally ‘SNS Addiction Disorder’) because addiction criteria, such as neglect of personal life, mental preoccupation, escapism, mood modifying experiences, tolerance, and concealing the addictive behavior, appear to be present in some people who use SNSs excessively [8].

Social Networking Sites are virtual communities where users can create individual public profiles, interact with real-life friends, and meet other people based on shared interests. SNSs are “web-based services that allow individuals to: (1) construct a public or semi-public profile within a bounded system, (2) articulate a list of other users with whom they share a connection, and (3) view and traverse their list of connections and those made by others within the system” [9]. The focus is placed on established networks, rather than on networking, which implies the construction of new networks. SNSs offer individuals the possibilities of networking and sharing media content, therefore embracing the main Web 2.0 attributes [10], against the framework of their respective structural characteristics.

In terms of SNS history, the first social networking site (*SixDegrees*) was launched in 1997, based on the idea that everybody is linked with everybody else via six degrees of separation [9], and initially referred to as the “small world problem” [11]. In 2004, the most successful current SNS, *Facebook*, was established as a closed virtual community for Harvard students. The site expanded very quickly and *Facebook* currently has more than 500 million users, of whom fifty percent log on to it every day. Furthermore, the overall time spent on *Facebook* increased by 566% from 2007 to 2008 [12]. This statistic alone indicates the exponential appeal of SNSs and also suggests a reason for a rise in potential SNS addiction. Hypothetically, the appeal of SNSs may be traced back to its reflection of today’s individualist culture. Unlike traditional virtual communities that emerged during the 1990s based on shared interests of their members [13], social networking sites are egocentric sites. It is the individual rather than the community that is the focus of attention [9].

Egocentrism has been linked to Internet addiction [14]. Supposedly, the egocentric construction of SNSs may facilitate the engagement in addictive behaviors and may thus serve as a factor that attracts people to using it in a potentially excessive way. This hypothesis is in line with the PACE Framework for the etiology of addiction specificity [15]. Attraction is one of the four key components that may predispose individuals to becoming addicted to specific behaviors or substances rather than specific others. Accordingly, due to their egocentric construction, SNSs allow individuals to present themselves positively that may “raise their spirits” (*i.e.*, enhance their mood state) because it is experienced as pleasurable. This may lead to positive experiences that can potentially cultivate and facilitate learning experiences that drive the development of SNS addiction.

A behavioral addiction such as SNS addiction may thus be seen from a biopsychosocial perspective [16]. Just like substance-related addictions, SNS addiction incorporates the experience of the ‘classic’ addiction symptoms, namely mood modification (*i.e.*, engagement in SNSs leads to a favourable change in emotional states), salience (*i.e.*, behavioral, cognitive, and emotional preoccupation with the SNS usage), tolerance (*i.e.*, ever increasing use of SNSs over time), withdrawal symptoms (*i.e.*, experiencing unpleasant physical and emotional symptoms when SNS use is restricted or stopped), conflict (*i.e.*, interpersonal and intrapsychic problems ensue because of SNS usage), and relapse (*i.e.*, addicts quickly revert back in their excessive SNS usage after an abstinence period).

Moreover, scholars have suggested that a combination of biological, psychological and social factors contributes to the etiology of addictions [16,17], that may also hold true for SNS addiction. From this it follows that SNS addiction shares a common underlying etiological framework with other substance-related and behavioral addictions. However, due to the fact that the engagement in SNSs is different in terms of the actual expression of (Internet) addiction (*i.e.*, pathological use of social networking sites rather than other Internet applications), the phenomenon appears worthy of individual consideration, particularly when considering the potentially detrimental effects of both substance-related and behavioral addictions on individuals who experience a variety of negative consequences because of their addiction [18].

To date, the scientific literature addressing the addictive qualities of social networks on the Internet is scarce. Therefore, with this literature review, it is intended to provide empirical insight into the emerging phenomenon of Internet social network usage and potential addiction by (1) outlining SNS usage patterns, (2) examining motivations for SNS usage, (3) examining personalities of SNS users, (4) examining negative consequences of SNSs, (5) exploring potential SNS addiction, and (6) exploring SNS addiction specificity and comorbidity.

## 2. Method

An extensive literature search was conducted using the academic database Web *of Knowledge* as well as *Google Scholar*. The following search terms as well as their derivatives were entered: social network, online network, addiction, compulsive, excessive, use, abuse, motivation, personality, and comorbidity. Studies were included if they: (i) included empirical data, (ii) made reference to usage patterns, (iii) motivations for usage, (iv) personality traits of users, (v) negative consequences of use, (vi) addiction, (vii) and/or comorbidity and specificity. A total of 43 empirical studies were identified from the literature, five of which specifically assessed SNS addiction.

## 3. Results

### 3.1. Usage

Social networking sites are seen as a ‘global consumer phenomenon’ and, as already noted, have experienced an exponential rise in usage within the last few years [12]. Of all Internet users, approximately one-third participate in SNSs and ten percent of the total time spent online is spent on SNSs [12]. In terms of usage, the results of the Parents and Teens 2006 Survey with a random sample of 935 participants in America revealed that 55% of youths used SNSs in that year [19]. The main reasons reported for this usage were staying in touch with friends (endorsed by 91%), and using them to make new friends (49%). This was more common among boys than girls. Girls preferred to use these sites in order to maintain contacts with actual friends rather than making new ones. Furthermore, half of the teenagers in this sample visited their SNS at least once a day which is indicative of the fact that in order to keep an attractive profile, frequent visits are necessary and this is a factor that facilitates potential excessive use [19]. Moreover, based on the results of consumer research, the overall usage of SNSs increased by two hours per month to 5.5 hours and active participation increased by 30% from 2009 to 2010 [5].

The findings of an online survey of 131 psychology students in the US [20] indicated that 78% used SNSs, and that 82% of males and 75% of females had SNS profiles. Of those, 57% used their SNS on a daily basis. The activities most often engaged in on SNSs were reading/responding to comments on their SNS page and/or posts to one’s wall (endorsed by 60%; the “wall” is a special profile feature in *Facebook*, where people can post comments, pictures, and links, that can be responded to), sending/responding to messages/invites (14%), and browsing friends’ profiles/walls/pages (13%; [20]). These results correspond with findings from a different study including another university student sample [21].

Empirical research has also suggested gender differences in SNS usage patterns. Some studies claim that men tend to have more friends on SNSs than women [22], whereas others have found the opposite [23]. In addition, men were found to take more risks with regards to disclosure of personal information [24,25]. Furthermore, one study reported that slightly more females used *MySpace* specifically (*i.e.*, 55% compared to 45% of males) [26].

Usage of SNSs has also been found to differ with regards to age group. A study comparing 50 teenagers (13–19 years) and the same number of older *MySpace* users (60 years and above) revealed that teenagers’ friends’ networks were larger and that their friends were more similar to themselves with regards to age [23]. Furthermore, older users’ networks were smaller and more dispersed age-wise. Additionally, teenagers made more use of *MySpace* web 2.0 features (*i.e.*, sharing video and music, and blogging) relative to older people [23].

With regards to how people react to using SNSs, a recent study [27] using psychophysiological measures (skin conductance and facial electromyography) found that social searching (*i.e.*, extracting information from friends’ profiles), was more pleasurable than social browsing (*i.e.*, passively reading newsfeeds) [27]. This finding indicates that the goal-directed activity of social searching may activate the appetitive system, which is related to pleasurable experience, relative to the aversive system [28]. On a neuroanatomical level, the appetitive system has been found to be activated in Internet game overusers and addicts [29,30], which may be linked back to a genetic deficiency in the addicts’ neurochemical reward system [31]. Therefore, the activation of the appetitive system in social network users who engage in social searching concurs with the activation of that system in people found to suffer from behavioral addictions. In order to establish this link for SNS specifically, further neurobiological research is required.

In reviewing SNS usage patterns, the findings of both consumer research and empirical research indicate that overall, regular SNS use has increased substantially over the last few years. This supports the availability hypothesis that where there is increased access and opportunity to engage in an activity (in this case SNSs), there is an increase in the numbers of people who engage in the activity [32]. Moreover, it indicates that individuals become progressively aware of this available supply and become more sophisticated with regards to their usage skills. These factors are associated with the pragmatics factor of addiction specificity etiology [15]. Pragmatics is one of the four key components of the addiction specificity model and it emphasizes access and habituation variables in the development of specific addictions. Therefore, the pragmatics of SNS usage appears to be a factor related to potential SNS addiction.

In addition to this, the findings of the presented studies indicate that compared to the general population, teenagers and students make most use of SNSs by utilizing the inherent Web 2.0 features. Additionally, there appear to be gender differences in usage, the specifics of which are only vaguely defined and thus require further empirical investigation. In addition, SNSs tend to be used mostly for social purposes of which extracting further information from friends’ pages appears particularly pleasurable. This, in turn, may be linked to the activation of the appetitive system, which indicates that engaging in this particular activity may stimulate the neurological pathways known to be related to addiction experience.

### 3.2. Motivations

Studies suggest that SNS usage in general, and *Facebook* in particular, differs as a function of motivation (*i.e.*, [33]). Drawing on uses and gratification theory, media are used in a goal-directed way for the purpose of gratification and need satisfaction [34] which have similarities with addiction. Therefore, it is essential to understand the motivations that underlie SNS usage. Persons with higher social identity (*i.e.*, solidarity to and conformity with their own social group), higher altruism (related to both, kin and reciprocal altruism) and higher telepresence (*i.e.*, feeling present in the virtual environment) tend to use SNSs because they perceive encouragement for participation from the social network [35]. Similarly, the results of a survey comprising 170 US university students indicated that social factors were more important motivations for SNS usage than individual factors [36]. More specifically, these participants’ interdependent self-construal (*i.e.*, the endorsement of collectivist cultural values), led to SNS usage that in turn resulted in higher levels of satisfaction, relative to independent self-construal, which refers to the adoption of individualist values. The latter were not related to motivations for using SNSs [36].

Another study by Barker [37] presented similar results, and found that collective self-esteem and group identification positively correlated with peer group communication via SNSs. Cheung, Chiu and Lee [38] assessed social presence (*i.e.*, the recognition that other persons share the same virtual realm, the endorsement of group norms, maintaining interpersonal interconnectivity and social enhancement with regards to SNS usage motivations). More specifically, they investigated the We-intention to use *Facebook* (*i.e.*, the decision to continue using a SNS together in the future). The results of their study indicated that We-intention positively correlated with the other variables [38].

Similarly, social reasons appeared as the most important motives for using SNSs in another study [20]. The following motivations were endorsed by the participating university student sample: keeping in touch with friends they do not see often (81%), using them because all their friends had accounts (61%), keeping in touch with relatives and family (48%), and making plans with friends they see often (35%). A further study found that a large majority of students used SNSs for the maintenance of offline relationships, whereas some preferred to use this type of Internet application for communication rather than face-to-face interaction [39].

The particular forms of virtual communication in SNSs include both asynchronous (*i.e.*, personal messages sent within the SNS) and synchronous modes (*i.e.*, embedded chat functions within the SNS) [40]. On behalf of the users, these communication modes require learning differential vocabularies, namely Internet language [41,42]. The idiosyncratic form of communication via SNSs is another factor that may fuel potential SNS addiction because communication has been identified as a component of the addiction specificity etiology framework [15]. Therefore, it can be hypothesized that users who prefer communication via SNSs (as compared to face-to-face communication) are more likely to develop an addiction to using SNSs. However, further empirical research is needed to confirm such a speculation.

Moreover, research suggests that SNSs are used for the formation and maintenance of different forms of social capital [43]. Social capital is broadly defined as *“the sum of the resources, actual or virtual, that accrue to an individual or a group by virtue of possessing a durable network of more or less institutionalized relationships of mutual acquaintance and recognition”* [44]. Putnam [45] differentiates bridging and bonding social capital from one another. Bridging social capital refers to weak connections between people that are based on information-sharing rather than emotional support. These ties are beneficial in that they offer a wide range of opportunities and access to broad knowledge because of the heterogeneity of the respective network’s members [46]. Alternatively, bonding social capital indicates strong ties usually between family members and close friends [45].

SNSs are thought to increase the size of potential networks because of the large number of possible weak social ties among members, which is enabled via the structural characteristics of digital technology [47]. Therefore, SNSs do not function as communities in the traditional sense. They do not include membership, shared influence, and an equal power allocation. Instead, they can be conceptualized as networked individualism, allowing the establishment of numerous self-perpetuating connections that appear advantageous for users [48]. This is supported by research that was carried out on a sample of undergraduate students [43]. More specifically, this study found that maintaining bridging social capital via participation in SNSs appeared to be beneficial for students with regards to potential employment opportunities in addition to sustaining ties with old friends. Overall, the benefits of bridging social capital formed via participation in SNSs appeared to be particularly advantageous for individuals with low-self esteem [49]. However, the ease of establishing and maintaining bridging social capital may become one of the reasons why people with low self-esteem are drawn to using SNSs in a potentially excessive manner. Lower self-esteem, in turn, has been linked to Internet addiction [50,51].

Furthermore, SNS usage has been found to differ between people and cultures. A recent study [52] including samples from the US, Korea and China demonstrated that the usage of different *Facebook* functions was associated with the creation and maintenance of either bridging or bonding social capital. People in the US used the ‘Communication’ function (*i.e.*, conversation and opinion sharing) in order to bond with their peers. However, Koreans and Chinese used ‘Expert Search’ (*i.e.*, searching for associated professionals online) and ‘Connection’ (*i.e.*, maintaining offline relationships) for the formation and sustaining of both bonding and bridging social capital [52]. These findings indicate that due to cultural differences in SNS usage patterns, it appears necessary to investigate and contrast SNS addiction in different cultures in order to discern both similarities and differences.

Additionally, the results of an online survey with a student convenience sample of 387 participants [53] indicated that several factors significantly predicted the intention to use SNSs as well as their actual usage. The identified predictive factors were (i) playfulness (*i.e.*, enjoyment and pleasure), (ii) the critical mass of the users who endorsed the technology, (iii) trust in the site, (iv) perceived ease of use, and (v) perceived usefulness. Moreover, normative pressure (*i.e.*, the expectations of other people with regards to one’s behavior) had a negative relationship with SNS usage. These results suggest that it is particularly the enjoyment associated with SNS use in a hedonic context (which has some similarities to addictions), as well as the recognition that a critical mass uses SNSs that motivates people to make use of those SNSs themselves [53].

Another study [54] used a qualitative methodology to investigate why teenagers use SNSs. Interviews were conducted with 16 adolescents aged 13 to 16 years. The results indicated that the sample used SNSs in order to express and actualize their identities either via self-display of personal information (which was true for the younger sample) or via connections (which was true for the older participants). Each of these motivations was found to necessitate a trade-off between potential opportunities for self-expression and risks with regards to compromising privacy on behalf of the teenagers [54].

A study by Barker [37] also suggested there may be differences in motivations for SNS use between men and women. Females used SNSs for communication with peer group members, entertainment and passing time, whereas men used it in an instrumental way for social compensation, learning, and social identity gratifications (*i.e.*, the possibility to identify with group members who share similar characteristics). Seeking friends, social support, information, and entertainment were found to be the most significant motivations for SNS usage in a sample of 589 undergraduate students [55]. In addition to this, endorsement of these motivations was found to differ across cultures. Kim *et al.* [55] found that Korean college students sought social support from already established relationships via SNSs, whereas American college students looked for entertainment. Similarly, Americans had significantly more online friends than Koreans, suggesting that the development and maintenance of social relationships on SNSs was influenced by cultural artefacts [55]. Furthermore, technology-relevant motivations were related to SNS use. The competence in using computer-mediated communication (*i.e.*, the motivation to, knowledge of, and efficacy in using electronic forms of communication) was found to be significantly associated with spending more time on *Facebook* and checking one’s wall significantly more often [33].

Overall, the results of these studies indicate that SNSs are predominantly used for social purposes, mostly related to the maintenance of established offline networks, relative to individual ones. In line with this, people may feel compelled to maintaining their social networks on the Internet which may lead to using SNSs excessively. The maintenance of already established offline networks itself can therefore be seen as an attraction factor, which according to Sussman *et al.* [15] is related to the etiology of specific addictions. Furthermore, viewed from a cultural perspective, it appears that motivations for usage differ between members of Asian and Western countries as well as between genders and age groups. However, in general, the results of the reported studies suggest that the manifold ties pursued online are indicative, for the most part, of bridging rather than bonding social capital. This appears to show that SNSs are primarily used as a tool for staying connected.

Staying connected is beneficial to such individuals because it offers them a variety of potential academic and professional opportunities, as well as access to a large knowledge base. As the users’ expectations of connectivity are met through their SNS usage, the potential for developing SNS addiction may increase as a consequence. This is in accordance with the expectation factor that drives the etiology of addiction to a specific behavior [15]. Accordingly, the supposed expectations and benefits of SNS use may go awry particularly for people with low self-esteem. They may feel encouraged to spend excessive amounts of time on SNSs because they perceive it as advantageous. This, in turn, may potentially develop into an addiction to using SNSs. Clearly, future research is necessary in order to establish this link empirically.

Moreover, there appear certain limitations to the studies presented. Many studies included small convenience samples, teenagers or university students as participants, therefore severely limiting the generalizability of findings. Thus, researchers are advised to take this into consideration and amend their sampling frameworks by using more representative samples and thus improve the external validity of the research.

### 3.3. Personality

A number of personality traits appear to be associated with the extent of SNS use. The findings of some studies (e.g., [33,56]) indicate that people with large offline social networks, who are more extroverted, and who have higher self-esteem, use *Facebook* for social enhancement, supporting the principle of ‘the rich get richer’. Correspondingly, the size of people’s online social networks correlates positively with life satisfaction and well-being [57], but does neither have an effect on the size of the offline network nor on emotional closeness to people in real life networks [58].

However, people with only a few offline contacts compensate for their introversion, low-self esteem, and low life-satisfaction by using *Facebook* for online popularity, thus corroborating the principle of ‘the poor get richer’ (*i.e.*, the social compensation hypothesis) [37,43,56,59]. Likewise, people higher in narcissistic personality traits tend to be more active on *Facebook* and other SNSs in order to present themselves favourably online because the virtual environment empowers them to construct their ideal selves [59–62]. The relationship between narcissism and *Facebook* activity may be related to the fact that narcissists have an imbalanced sense of self, fluctuating between grandiosity with regards to explicit agency and low self-esteem concerning implicit communion and vulnerability [63,64]. Narcissistic personality, in turn, has been found to be associated with addiction [65]. This finding will be discussed in more detail in the section on addiction.

Moreover, it appears that people with different personality traits differ in their usage of SNSs [66] and prefer to use distinct functions of *Facebook* [33]. People high in extraversion and openness to experience use SNSs more frequently, with the former being true for mature and the latter for young people [66]. Furthermore, extraverts and people open to experiences are members of significantly more groups on *Facebook,* use socializing functions more [33], and have more *Facebook* friends than introverts [67], which delineates the former’s higher sociability in general [68]. Introverts, on the other hand, disclose more personal information on their pages [67]. Additionally, it appears that particularly shy people spend large amounts of time on *Facebook* and have large amounts of friends on this SNS [69]. Therefore, SNSs may appear beneficial for those whose real-life networks are limited because of the possibility of easy access to peers without the demands of real-life proximity and intimacy. This ease of access entails a higher time commitment for this group, which may possibly result in excessive and/or potentially addictive use.

Likewise, men with neurotic traits use SNSs more frequently than women with neurotic traits [66]. Furthermore, neurotics (in general) tend to use *Facebook’s* wall function, where they can receive and post comments, whereas people with low neuroticism scores prefer posting photos [33]. This may be due to the neurotic individual’s greater control over emotional content with regards to text-based posts rather than visual displays [33]. However, another study [67] found the opposite, namely that people scoring high on neuroticism were more inclined to post their photographs on their page. In general, the findings for neuroticism imply that those scoring high on this trait disclose information because they seek self-assurance online, whereas those scoring low are emotionally secure and thus share information in order to express themselves [67]. High self-disclosure on SNSs, in turn, was found to positively correlate with measures of subjective well-being [57]. It remains questionable whether this implies that low self-disclosure on SNSs may be related to higher risk for potential addiction. By disclosing more personal information on their pages, users put themselves at risk for negative feedback, which has been linked to lower well-being [70]. Therefore, the association between self-disclosure on SNSs and addiction needs to be addressed empirically in future studies.

With regards to agreeableness, it was found that females scoring high on this trait upload significantly more pictures than females scoring low, with the opposite being true for males [67]. In addition to this, people with high conscientiousness were found to have significantly more friends and to upload significantly less pictures than those scoring low on this personality trait [67]. An explanation for this finding may be that conscientious people tend to cultivate their online and offline contacts more without the necessity to share too much personal information publicly.

Overall, the results of these studies suggest that extraverts use SNSs for social enhancement, whereas introverts use it for social compensation, each of which appears to be related to greater SNS usage. With regards to addiction, both groups could potentially develop addictive tendencies for different reasons, namely social enhancement and social compensation. In addition, the dissimilar findings of studies with regards to the number of friends introverts have online deserve closer scrutiny in future research. The same applies for the results with regards to neuroticism. On the one hand, neurotics use SNSs frequently. On the other hand, studies indicate different usage preferences for people who score high on neuroticism, which calls for further investigation. Furthermore, the structural characteristics of these Internet applications, (*i.e.*, their egocentric construction) appear to allow favourable self-disclosure, which draws narcissists to use it. Finally, agreeableness and conscientiousness appear to be related to the extent of SNS usage. Higher usage associated with narcissistic, neurotic, extravert and introvert personality characteristics may implicate that each of these groups is particularly at risk for developing an addiction to using SNSs.

### 3.4. Negative Correlates

Some studies have highlighted a number of potential negative correlates of extensive SNS usage. For instance, the results of an online survey of 184 Internet users indicated that people who use SNS more in terms of time spent on usage were perceived to be less involved with their real life communities [71]. This is similar to the finding that people who do not feel secure about their real-life connections to peers and thus have a negative social identity tend to use SNSs more in order to compensate for this [37]. Moreover, it seems that the nature of the feedback from peers that is received on a person’s SNS profile determines the effects of SNS usage on wellbeing and self-esteem.

More specifically, Dutch adolescents aged 10 to 19 years who received predominantly negative feedback had low self-esteem which in turn led to low wellbeing [70]. Given that people tend to be disinhibited when they are online [72], giving and receiving negative feedback may be more common on the Internet than in real life. This may entail negative consequences particularly for people with low self-esteem who tend to use SNSs as compensation for real-life social network paucity because they are dependent upon the feedback they receive via these sites [43]. Therefore, potentially, people with lower self-esteem are a population at risk for developing an addiction to using SNSs.

According to a more recent study assessing the relationships between *Facebook* usage and academic performance in a sample of 219 university students [73], *Facebook* users had lower Grade Point Averages and spent less time studying than students who did not use this SNS. Of the 26% of students reporting an impact of their usage on their lives, three-quarters (74%) claimed that it had a negative impact, namely procrastination, distraction, and poor time-management. A potential explanation for this may be that students who used the Internet to study may have been distracted by simultaneous engagement in SNSs, implying that this form of multitasking is detrimental to academic achievement [73].

In addition to this, it appears that the usage of *Facebook* may in some circumstances have negative consequences for romantic relationships. The disclosure of rich private information on one’s *Facebook* page including status updates, comments, pictures, and new friends, can result in jealous cyberstalking [74], including interpersonal electronic surveillance (IES; [75]) by one’s partner. This was reported to lead to jealousy [76,77] and, in the most extreme cases, divorce and associated legal action [78].

These few existent studies highlight that in some circumstances, SNS usage can lead to a variety of negative consequences that imply a potential decrease in involvement in real-life communities and worse academic performance, as well as relationship problems. Reducing and jeopardizing academic, social and recreational activities are considered as criteria for substance dependence [18] and may thus be considered as valid criteria for behavioral addictions [79], such as SNS addiction. In light of this, endorsing these criteria appears to put people at risk for developing addiction and the scientific research base outlined in the preceding paragraphs supports the potentially addictive quality of SNSs.

Notwithstanding these findings, due to the lack of longitudinal designs used in the presented studies, no causal inferences can be drawn with regards to whether the excessive use of SNSs is the causal factor for the reported negative consequences. Moreover, potential confounders need to be taken into consideration. For instance, the aspect of university students’ multi-tasking when studying appears to be an important factor related to poor academic achievement. Moreover, pre-existent relationship difficulties in the case of romantic partners may potentially be exacerbated by SNS use, whereas the latter does not necessarily have to be the primary driving force behind the ensuing problems. Nevertheless, the findings support the idea that SNSs are used by some people in order to cope with negative life events. Coping, in turn, has been found to be associated with both substance dependence and behavioral addictions [80]. Therefore, it appears valid to claim that there is a link between dysfunctional coping (*i.e.*, escapism and avoidance) and excessive SNS use/addiction. In order to substantiate this conjecture and to more fully investigate the potential negative correlates associated with SNS usage, further research is needed.

### 3.5. Addiction

Researchers have suggested that the excessive use of new technologies (and especially online social networking) may be particularly addictive to young people [81]. In accordance with the biopsychosocial framework for the etiology of addictions [16] and the syndrome model of addiction [17], it is claimed that those people addicted to using SNSs experience symptoms similar to those experienced by those who suffer from addictions to substances or other behaviors [81]. This has significant implications for clinical practice because unlike other addictions, the goal of SNS addiction treatment cannot be total abstinence from using the Internet *per se* since the latter is an integral element of today’s professional and leisure culture. Instead, the ultimate therapy aim is controlled use of the Internet and its respective functions, particularly social networking applications, and relapse prevention using strategies developed within cognitive-behavioral therapies [81].

In addition to this, scholars have hypothesized that young vulnerable people with narcissistic tendencies are particularly prone to engaging with SNSs in an addictive way [65]. To date, only three empirical studies have been conducted and published in peer-reviewed journals that have specifically assessed the addictive potential of SNSs [82–84]. In addition to this, two publicly available Master’s theses have analyzed the SNS addiction and will be presented subsequently for the purpose of inclusiveness and the relative lack of data on the topic [85,86]. In the first study [83], 233 undergraduate university students (64% females, mean age = 19 years, *SD* = 2 years) were surveyed using a prospective design in order to predict high level use intentions and actual high-level usage of SNSs via an extended model of the theory of planned behavior (TPB; [87]). High-level usage was defined as using SNSs at least four times per day. TPB variables included measures of intention for usage, attitude, subjective norm, and perceived behavioral control (PBC). Furthermore, self-identity (adapted from [88]), belongingness [89], as well as past and potential future usage of SNSs were investigated. Finally, addictive tendencies were assessed using eight questions scored on Likert scales (based on [90]).

One week after completion of the first questionnaire, participants were asked to indicate on how many days during the last week they had visited SNSs at least four times a day. The results of this study indicated that past behavior, subjective norm, attitude, and self-identity significantly predicted both behavioral intention as well as actual behavior. Additionally, addictive tendencies with regards to SNS use were significantly predicted by self-identity and belongingness [83]. Therefore, those who identified themselves as SNS users and those who looked for a sense of belongingness on SNSs appeared to be at risk for developing an addiction to SNSs.

In the second study [82], an Australian university student sample of 201 participants (76% female, mean age = 19, *SD* = 2) was drawn upon in order to assess personality factors via the short version of the NEO Personality Inventory (NEO-FFI; [91]), the Self-Esteem Inventory (SEI; [92]), time spent using SNSs, and an Addictive Tendencies Scale (based on [90,93]). The Addictive Tendencies Scale included three items measuring salience, loss of control, and withdrawal. The results of a multiple regression analysis indicated that high extraversion and low conscientiousness scores significantly predicted both addictive tendencies and the time spent using an SNS. The researchers suggested that the relationship between extraversion and addictive tendencies could be explained by the fact that using SNSs satisfies the extraverts’ need to socialize [82]. The findings with regards to lack of conscientiousness appear to be in line with previous research on the frequency of general Internet use in that people who score low on conscientiousness tend to use the Internet more frequently than those who score high on this personality trait [94].

In the third study, Karaiskos *et al.* [84] report the case of a 24-year old female who used SNS to such an extent that her behavior significantly interfered with her professional and private life. As a consequence, she was referred to a psychiatric clinic. She used *Facebook* excessively for at least five hours a day and was dismissed from her job because she continuously checked her SNS instead of working. Even during the clinical interview, she used her mobile phone to access *Facebook*. In addition to excessive use that led to significant impairment in a variety of areas in the woman’s life, she developed anxiety symptoms as well as insomnia, which suggestively points to the clinical relevance of SNS addiction. Such extreme cases have led to some researchers to conceptualize SNS addiction as Internet spectrum addiction disorder [84]. This indicates that first, SNS addiction can be classified within the larger framework of Internet addictions, and second, that it is a specific Internet addiction, alongside other addictive Internet applications such as Internet gaming addiction [95], Internet gambling addiction [96], and Internet sex addiction [97].

In the fourth study [85], SNS game addiction was assessed via the Internet Addiction Test [98] using 342 Chinese college students aged 18 to 22 years. In this study, SNS game addiction referred specifically to being addicted to the SNS game *Happy Farm*. Students were defined as addicted to using this SNS game when they endorsed a minimum of five out of eight total items of the IAT. Using this cut-off, 24% of the sample were identified as addicted [85].

Moreover, the author investigated gratifications of SNS game use, loneliness [99], leisure boredom [100], and self esteem [101]. The findings indicated that there was a weak positive correlation between loneliness and SNS game addiction and a moderate positive correlation between leisure boredom and SNS game addiction. Moreover, the gratifications “inclusion” (in a social group) and “achievement” (in game), leisure boredom, and male gender significantly predicted SNS game addiction [85].

In the fifth study [86], SNS addiction was assessed in a sample of 335 Chinese college students aged 19 to 28 years using Young’s Internet Addiction Test [98] modified to specifically assess the addiction to a common Chinese SNS, namely *Xiaonei.com*. Users were classified as addicted when they endorsed five or more of the eight addiction items specified in the IAT. Moreover, the author assessed loneliness [99], user gratifications (based on the results of a previous focus group interview), usage attributes and patterns of SNS website use [86].

The results indicated that of the total sample, 34% were classified as addicted. Moreover, loneliness significantly and positively correlated with frequency and session length of using *Xiaonei.com* as well as SNS addiction. Likewise, social activities and relationship building were found to predict SNS addiction [86].

Unfortunately, when viewed from a critical perspective, the quantitative studies reviewed here suffer from a variety of limitations. Initially, the mere assessment of addiction tendencies does not suffice to demarcate real pathology. In addition, the samples were small, specific, and skewed with regards to female gender. This may have led to the very high addiction prevalence rates (up to 34%) reported [86]. Clearly, it needs to be ensured that rather than assessing excessive use and/or preoccupation, addiction specifically needs to be assessed.

Wilson *et al.*’s study [82] suffered from endorsing only three potential addiction criteria which is not sufficient for establishing addiction status clinically. Similarly, significant impairment and negative consequences that discriminate addiction from mere abuse [18] were not assessed in this study at all. Thus, future studies have great potential in addressing the emergent phenomenon of addiction to using social networks on the Internet by means of applying better methodological designs, including more representative samples, and using more reliable and valid addiction scales so that current gaps in empirical knowledge can be filled.

Furthermore, research must address the presence of specific addiction symptoms beyond negative consequences. These might be adapted from the DSM-IV TR criteria for substance dependence [18] and the ICD-10 criteria for a dependence syndrome [102], including (i) tolerance, (ii) withdrawal, (iii) increased use, (iv) loss of control, (v) extended recovery periods, (vi) sacrificing social, occupational and recreational activities, and (vii) continued use despite of negative consequences. These have been found to be adequate criteria for diagnosing behavioral addictions [79] and thus appear sufficient to be applied to SNS addiction. In order to be diagnosed with SNS addiction, at least three (but preferably more) of the above mentioned criteria should be met in the same 12-month period and they must cause significant impairment to the individual [18].

In light of this qualitative case study, it appears that from a clinical perspective, SNS addiction is a mental health problem that may require professional treatment. Unlike the quantitative studies, the case study emphasizes the significant individual impairment that is experienced by individuals that spans a variety of life domains, including their professional life as well as their psychosomatic condition. Future researchers are therefore advised to not only investigate SNS addiction in a quantitative way, but to further our understanding of this new mental health problem by analyzing cases of individuals who suffer from excessive SNS usage.

### 3.6. Specificity and Comorbidity

It appears essential to pay adequate attention to (i) the specificity of SNS addiction and (ii) potential comorbidity. Hall *et al.* [103] outline three reasons why it is necessary to address comorbidity between mental disorders, such as addictions. First, a large number of mental disorders feature additional (sub)clinical problems/disorders. Second, comorbid conditions must be addressed in clinical practice in order to improve treatment outcomes. Third, specific prevention programs may be developed which incorporate different dimensions and treatment modalities that particularly target associated mental health problems. From this it follows that assessing the specificity and potential comorbidities of SNS addiction is important. However, to date, research addressing this topic is virtually non-existent. There has been almost no research on the co-occurrence of SNS addiction with other types of addictive behavior, mainly because there have been so few studies examining SNS addiction as highlighted in the previous section. However, based on the small empirical base, there are a number of speculative assumptions that can be made about co-addiction co-morbidity in relation to SNS addiction.

Firstly, for some individuals, their SNS addiction takes up such a large amount of available time that it is highly unlikely that it would co-occur with other behavioral addictions unless the other behavioral addiction(s) can find an outlet via social networking sites (e.g., gambling addiction, gaming addiction). Put simply, there would be little face validity in the same individual being, for example, both a workaholic and a social networking addict, or an exercise addict and a social networking addict, mainly because the amount of daily time available to engage in two behavioral addictions simultaneously would be highly unlikely. Still, it is necessary to pinpoint the respective addictive behaviors because some of these behaviors may in fact co-occur. In one study that included a clinical sample diagnosed with substance dependencies, Malat and colleagues [104] found that 61% pursued at least one and 31% engaged in two or more problematic behaviors, such as overeating, unhealthy relationships and excessive Internet use. Therefore, although a simultaneous addiction to behaviors such as working and using SNS is relatively unlikely, SNS addiction may potentially co-occur with overeating and other excessive sedentary behaviors.

Thus, secondly, it is theoretically possible for a social networking addict to have an additional drug addiction, as it is perfectly feasible to engage in both a behavioral and chemical addiction simultaneously [16]. It may also make sense from a motivational perspective. For instance, if one of the primary reasons social network addicts are engaging in the behavior is because of their low self-esteem, it makes intuitive sense that some chemical addictions may serve the same purpose. Accordingly, studies suggest that the engagement in addictive behaviors is relatively common among persons who suffer from substance dependence. In one study, Black *et al.* [105] found that 38% of problematic computer users in their sample had a substance use disorder in addition to their behavioral problems/addiction. Apparently, research indicates that some persons who suffer from Internet addiction experience other addictions at the same time.

Of a patient sample including 1,826 individuals treated for substance addictions (mainly cannabis addiction), 4.1% were found to suffer from Internet addiction [106]. Moreover, the findings of further research [107] indicated that Internet addiction and substance use experience in adolescents share common family factors, namely higher parent-adolescent conflict, habitual alcohol use of siblings, perceived parents’ positive attitude to adolescent substance use, and lower family functioning. Moreover, Lam *et al.* [108] assessed Internet addiction and associated factors in a sample of 1,392 adolescents aged 13–18 years. In terms of potential comorbidity, they found that drinking behavior was a risk factor for being diagnosed with Internet addiction using the Internet Addiction Test [109]. This implies that potentially, alcohol abuse/dependence can be associated with SNS addiction. Support for this comes from Kuntsche *et al.* [110]. They found that in Swiss adolescents, the expectancy of social approval was associated with problem drinking. Since SNSs are inherently social platforms that are used by people for social purposes, it appears reasonable to deduce that there may indeed be people who suffer from comorbid addictions, namely SNS addiction and alcohol dependence.

Thirdly, it appears that there may be a relationship between SNS addiction specificity and personality traits. Ko *et al.* [111] found that Internet addiction (IA) was predicted by high novelty seeking (NS), high harm avoidance (HA), and low reward dependence (RD) in adolescents. Those adolescents who were addicted to the Internet and who had experience of substance use scored significantly higher on NS and lower on HA than the IA group. Therefore, it appears that HA particularly impacts Internet addiction specificity because high HA discriminates Internet addicts from individuals who are not only addicted to the Internet, but who use substances. Therefore, it seems plausible to hypothesize that persons with low harm avoidance are in danger of developing comorbid addictions to SNSs and substances. Accordingly, research needs to address this difference specifically for those who are addicted to using SNSs in order to demarcate this potential disorder from comorbid conditions.

In addition to this, it seems reasonable to specifically address the respective activities people can engage in on their SNS. There have already been a number of researchers who have begun to examine the possible relationship between social networking and gambling [112–116], and social networking and gaming [113,116,117]. All of these writings have noted how the social networking medium can be used for gambling and/or gaming. For instance, online poker applications and online poker groups on social networking sites are among the most popular [115], and others have noted the press reports surrounding addiction to social networking games such as *Farmville* [117]. Although there have been no empirical studies to date examining addiction to gambling or gaming via social networking, there is no reason to suspect that those playing in the social networking medium are any less likely than those playing other online or offline media to become addicted to gambling and/or gaming.

Synoptically, addressing the specificity of SNS addiction and comorbidities with other addictions is necessary for (i) comprehending this disorder as distinct mental health problem while (ii) paying respect to associated conditions, which will (iii) aid treatment and (iv) prevention efforts. From the reported studies, it appears that the individual’s upbringing and psychosocial context are influential factors with regards to potential comorbidity between Internet addiction and substance dependence, which is supported by scientific models of addictions and their etiology [16,17]. Moreover, alcohol and cannabis dependence were outlined as potential co-occurring problems. Nonetheless, apart from this, the presented studies do not specifically address the discrete relationships between particular substance dependencies and individual addictive behaviors, such as addiction to using SNSs. Therefore, future empirical research is needed in order to shed more light upon SNS addiction specificity and comorbidity.

## 4. Discussion and Conclusions

The aim of this literature review was to present an overview of the emergent empirical research relating to usage of and addiction to social networks on the Internet. Initially, SNSs were defined as virtual communities offering their members the possibility to make use of their inherent Web 2.0 features, namely networking and sharing media content. The history of SNSs dates back to the late 1990s, suggesting that they are not as new as they may appear in the first place. With the emergence of SNSs such as *Facebook*, overall SNS usage has accelerated in such a way that they are considered a global consumer phenomenon. Today, more than 500 million users are active participants in the *Facebook* community alone and studies suggest that between 55% and 82% of teenagers and young adults use SNSs on a regular basis. Extracting information from peers’ SNS pages is an activity that is experienced as especially enjoyable and it has been linked with the activation of the appetitive system, which in turn is related to addiction experience.

In terms of sociodemographics, the studies presented indicate that overall, SNS usage patterns differ. Females appear to use SNS in order to communicate with members of their peer group, whereas males appear to use them for the purposes of social compensation, learning, and social identity gratifications [37]. Furthermore, men tend to disclose more personal information on SNS sites relative to women [25,118]. Also, more women were found to use *MySpace* specifically relative to men [26]. Moreover, usage patterns were found to differ between genders as a function of personality. Unlike women with neurotic traits, men with neurotic traits were found to be more frequent SNS users [66]. In addition to this, it was found that males were more likely to be addicted to SNS games specifically relative to females [85]. This is in line with the finding that males in general are a population at risk for developing an addiction to playing online games [95].

The only study that assessed age differences in usage [23] indicated that the latter in fact varies as a function of age. Specifically, “silver surfers” (*i.e.*, those over the age of 60 years) have a smaller circle of online friends that differs in age relative to younger SNS users. Based on the current empirical knowledge that has predominantly assessed young teenage and student samples, it appears unclear whether older people use SNSs excessively and whether they potentially become addicted to using them. Therefore, future research must aim at filling this gap in knowledge.

Next, the motivations for using SNSs were reviewed on the basis of needs and gratifications theory. In general, research suggests that SNSs are used for social purposes. Overall, the maintenance of connections to offline network members was emphasized rather than the establishment of new ties. With regards to this, SNS users sustain bridging social capital through a variety of heterogeneous connections to other SNS users. This appeared to be beneficial for them with regards to sharing knowledge and potential future possibilities related to employment and related areas. In effect, the knowledge that is available to individuals via their social network can be thought of as “collective intelligence” [119].

Collective intelligence extends the mere idea of shared knowledge because it is not restricted to knowledge shared by all members of a particular community. Instead, it denotes the aggregation of each individual member’s knowledge that can be accessed by other members of the respective community. In this regard, the pursuit of weak ties on SNSs is of great benefit and thus coincides with the satisfaction of the members’ needs. At the same time, it is experienced as gratifying. Therefore, rather than seeking emotional support, individuals make use of SNSs in order to communicate and stay in touch not only with family and friends, but also with more distant acquaintances, therefore sustaining weak ties with potentially advantageous environments. The benefits of large online social networks may potentially lead people to excessively engage in using them, which, in turn, may purport addictive behaviors.

As regards personality psychology, certain personality traits were found to be associated with higher usage frequency that may be associated with potential abuse and/or addiction. Of those, extraversion and introversion stand out because each of these is related to more habitual participation in social networks on the Internet. However, the motivations of extraverts and introverts differ in that extraverts enhance their social networks, whereas introverts compensate for the lack of real life social networks. Presumably, the motivations for higher SNS usage of people who are agreeable and conscientious may be related to those shared by extraverts, indicating a need for staying connected and socializing with their communities. Nevertheless, of those, high extraversion was found to be related to potential addiction to using SNS, in accordance with low conscientiousness [82].

The dissimilar motivations for usage found for members scoring high on the respective personality trait can inform future research into potential addiction to SNSs. Hypothetically, people who compensate for scarce ties with their real life communities may be at greater risk to develop addiction. In effect, in one study, addictive SNS usage was predicted by looking for a sense of belongingness in this community [83], which supports this conjecture. Presumably, the same may hold true for people who score high on neuroticism and narcissism, assuming that members of both groups tend to have low self-esteem. This supposition is informed by research indicating that people use the Internet excessively in order to cope with everyday stressors [120,121]. This may serve as a preliminary explanation for the findings regarding the negative correlates that were found to be associated with more frequent SNS usage.

Overall, the engagement in particular activities on SNSs, such as social searching, and the personality traits that were found to be associated with greater extents of SNS usage may serve as an anchor point for future studies in terms of defining populations who are at risk for developing addiction to using social networks on the Internet. Furthermore, it is recommended that researchers assess factors that are specific to SNS addiction, including the pragmatics, attraction, communication and expectations of SNS use because these may predict the etiology of SNS addiction as based on the addiction specificity etiology framework [15]. Due to the scarcity of research in this domain with a specific focus on SNS addiction specificity and comorbidity, further empirical research is necessary. Moreover, researchers are encouraged to pay close attention to the different motivations of introverts and extraverts because each of those appears to be related to higher usage frequency. What is more, investigating the relationship of potential addiction with narcissism seems to be a fruitful area for empirical research. In addition to this, motivations for usage as well as a wider variety of negative correlates related to excessive SNS use need to be addressed.

In addition to the above mentioned implications and suggestions for future research, specific attention needs to be paid to selecting larger samples which are representative of a broader population in order to increase the respective study’s external validity. The generalizability of results is essential in order to demarcate populations at risk for developing addiction to SNSs. Similarly, it appears necessary to conduct further psychophysiological studies in order to assess the phenomenon from a biological perspective. Furthermore, clear-cut and validated addiction criteria need to be assessed. It is insufficient to limit studies into addiction to assessing just a few criteria. The demarcation of pathology from high frequency and problematic usage necessitates adopting frameworks that have been established by the international classification manuals [18,102]. Moreover, in light of clinical evidence and practice, it appears essential to pay attention to the significant impairment that SNS addicts experience in a variety of life domains as a consequence of their abusive and/or addictive behaviors.

Similarly, the results of data based on self-reports are not sufficient for diagnosis because research suggests that they may be inaccurate [122]. Conceivably, self-reports may be supplemented with structured clinical interviews [123] and further case study evidence as well as supplementary reports from the users’ significant others. In conclusion, social networks on the Internet are iridescent Web 2.0 phenomena that offer the potential to become part of, and make use of, collective intelligence. However, the latent mental health consequences of excessive and addictive use are yet to be explored using the most rigorous scientific methods.
